# Clinical significance of serum calcium-phosphorus product, parathyroid hormone, vitamin K2 in chronic kidney disease patients with coronary artery calcification

**DOI:** 10.1080/0886022X.2025.2610796

**Published:** 2026-01-21

**Authors:** Chengbo Chen, Tianbao Chen, Youbang Xu

**Affiliations:** Department of Cardiology, Quanzhou First Affiliated Hospital of Fujian Medical University, Quanzhou, Fujian, China

**Keywords:** Calcium-phosphorus product, parathyroid hormone, vitamin K2, CKD, kidney

## Abstract

Coronary artery calcification (CAC) is a major cardiovascular complication in chronic kidney disease (CKD) driven by disrupted calcium-phosphorus metabolism, secondary hyperparathyroidism, and vitamin K2 deficiency. Although CAC predicts cardiovascular events, the roles of calcium-phosphorus product (Ca × P), parathyroid hormone (PTH), and vitamin K2 in CAC progression and diagnosis remain underexplored. This study examines clinical and biochemical factors linked to CAC in CKD and evaluates the diagnostic potential of these biomarkers individually and in combination. This retrospective analysis included 368 CKD patients divided into CAC (*n* = 216) and non-CAC (*n* = 152) groups based on coronary computed tomography angiography. Serum Ca × P, PTH, and vitamin K2 levels were assessed *via* biochemical assays, and CAC severity was quantified using Agatston scores. Correlations between biomarkers and CAC scores were analyzed, and ROC analysis determined the diagnostic performance of individual and combined biomarkers. CAC patients exhibited higher Ca × P and PTH levels and lower vitamin K2 levels than non-CAC patients. Ca × P and PTH positively correlated, while both inversely correlated with vitamin K2. CAC severity correlated positively with Ca × P and PTH but negatively with vitamin K2. The combined biomarker model achieved an AUC of 0.88, with 82.41% sensitivity and 77.63% specificity, surpassing individual markers. Ca × P, PTH, and vitamin K2 significantly correlated with CAC pathogenesis and severity in CKD. Their combination could potentially improve diagnostic accuracy, offering a promising approach for early detection and management of CAC in CKD patients.

## Introduction

Chronic kidney disease (CKD) is a global health burden characterized by progressive deterioration of kidney function and increased morbidity and mortality. Among its various complications, cardiovascular disease remains the leading cause of death in CKD patients, with coronary artery calcification (CAC) playing a pivotal role in cardiovascular pathology [1,2]. CAC, defined as the deposition of calcium in coronary arteries, is a key indicator of atherosclerotic disease and strongly correlates with cardiovascular events, particularly in CKD patients who are at higher risk due to their disrupted mineral metabolism [3,4]. Despite advances in CKD management, addressing the cardiovascular complications of CKD remains a pressing challenge.

CKD patients often exhibit abnormalities in calcium and phosphorus metabolism, parathyroid hormone (PTH) regulation, and vitamin K2 levels, all of which are implicated in the pathogenesis of vascular calcification [5,6]. Elevated calcium-phosphorus product (Ca × P) and PTH levels are widely recognized as contributors to vascular calcification through their promotion of osteogenic differentiation of vascular smooth muscle cells [7,8]. Elevated Ca × P levels lead to phosphate accumulation in arterial walls, inducing matrix mineralization and vascular stiffness [9,10]. Conversely, vitamin K2 is a critical cofactor for the activation of matrix Gla-protein (MGP), a potent inhibitor of vascular calcification, and deficiencies in vitamin K2 are associated with increased vascular calcification and cardiovascular events [5,6, 9,10]. The interplay between these metabolic factors exacerbates vascular calcification, particularly in CKD patients in advanced stages.

Although CAC is highly prevalent among CKD patients, its diagnosis and management remain challenging. Coronary computed tomography angiography (CTA) is the gold standard for CAC assessment, providing noninvasive quantification of coronary calcification [11]. However, identifying the biochemical pathways and biomarkers involved in CAC progression and their role in early detection and management requires further investigation. Studies suggest that combining multiple biomarkers, such as Ca × P, PTH, and vitamin K2, may improve diagnostic accuracy and prediction of CAC [12,13]. However, comprehensive analyses of these biomarkers in CKD populations are limited, and their role in assessing CAC severity and progression is not fully understood [13,14].

This study aimed to explore the clinical and biochemical profiles of CKD patients with and without CAC, focusing on the associations between serum Ca × P, PTH, and vitamin K2 levels. Additionally, we investigated the relationships between these biomarkers and CAC severity, assessing their diagnostic potential individually and in combination. By elucidating these interrelationships, this research provides critical insights into the pathophysiology of CAC in CKD and identifies potential strategies for improving risk stratification and clinical management. These findings build on previous work and aim to fill gaps in understanding CAC pathogenesis and its biochemical markers in CKD [15].

## Methods

### Study design and participants

This study retrospectively reviewed 368 patients diagnosed with CKD who were admitted to our department and underwent coronary CTA. The participants were divided into two groups: 216 patients with CAC and 152 without CAC (NCAC). The inclusion criteria for the study were: (1) age ≥18 years; (2) confirmed diagnosis of CKD; and (3) complete medical records with CTA performed in our hospital. Exclusion criteria included: history of parathyroidectomy; comorbid autoimmune diseases such as systemic lupus erythematosus or rheumatoid arthritis; malignancies; acute cardiovascular or cerebrovascular events; acute infectious diseases; or use of medications affecting calcium-phosphorus metabolism (e.g. calcium or phosphorus binders) during the study period. The study was approved by Quanzhou First Affiliated Hospital of Fujian Medical University, and written informed consent was derived from the participants.

### Diagnosis and staging of CKD

CKD was diagnosed according to the Kidney Disease Improving Global Outcomes diagnostic criteria. Patients met one of the following criteria for at least three months: (1) presence of one or more markers of kidney damage, such as albuminuria (albumin excretion rate ≥30 mg/24 h or urine albumin-to-creatinine ratio ≥3 mg/mmol), abnormal urine sediment, tubulointerstitial disease, structural abnormalities on imaging, pathological changes in kidney biopsies, or a history of kidney transplantation; or (2) glomerular filtration rate (GFR) ≤60 mL/min·1.73 m^2^, measured persistently for at least three months. Estimated GFR (eGFR) was calculated using the CKD-EPI (Chronic Kidney Disease Epidemiology Collaboration) formula, and CKD staging was categorized as follows: stage 1 (eGFR ≥90 mL/min·1.73 m^2^), stage 2 (eGFR 60–89 mL/min·1.73 m^2^), stage 3 (eGFR 30–59 mL/min·1.73 m^2^), stage 4 (eGFR 15–29 mL/min·1.73 m^2^), and stage 5 (eGFR <15 mL/min·1.73 m^2^).

### Definition of smoking and drinking

Smoking is defined as the act of inhaling and exhaling smoke from burned tobacco or nicotine-containing substances. Drinking is defined as the consumption of alcoholic beverages, quantified by ethanol intake.

### PTH and vitamin K2 measurements

PTH was measured using the chemiluminescent immunoassay method performed on the Dxi 800 automated chemiluminescent immunoassay analyzer manufactured by Beckman Coulter, Inc. Vitamin K2 was quantified using high-performance liquid chromatography conducted on the 1260 Infinity II HPLC system produced by Agilent Technologies, Inc.

### Measurments of creatinine, urine protein, serum uric acid

The levels of creatinine, urine protein and serum uric acid were determined by automated biochemical analyzers (BS-2800, Mindray, Shanghai, China).

### Coronary CTA and CAC scoring

All participants underwent 64-slice spiral CTA to assess CAC. Before the scan, patients’ ventricular rates were controlled to optimize imaging conditions. The Agatston method was used to calculate CAC scores. Calcified plaques were defined as lesions with a minimum area of ≥1 mm^2^ and a CT density of ≥130 Hounsfield units (HU). The calcification score was calculated as the product of the peak CT density and the plaque area (mm^2^), summing scores for all slices to obtain a total CAC score. The four main coronary artery branches—left main, left anterior descending, left circumflex, and right coronary artery—were scored separately, and their sum was recorded as the total CAC score. When the total score is greater than 0, it is defined as “presence of CAC” (included in the CAC group); when the total score is equal to 0, it is defined as “absence of CAC” (included in the NCAC group).

### Statistical analysis

Demographic and clinical data were analyzed to identify differences between the CAC and NCAC groups. Continuous variables were expressed as mean ± standard deviation (SD) and Shapiro-Wilk test was used first to test the normality. Unpaired *t*-test was used when the data met normality otherwise Mann-Whitney *U* test was selected. Categorical variables were reported as frequencies (percentages) and compared using Fisher’s exact test or chi-square test. Correlation analyses (Pearson and Spearman) were conducted to evaluate associations between biochemical markers and CAC scores. To determine the optimal cutoff value, the receiver operating characteristic (ROC) curve was analyzed using the Youden index (sensitivity + specificity − 1) to assess the diagnostic value of individual and combined biomarkers for CAC, and optimal cutoff values corresponding to the maximum Youden index was selected as the diagnostic threshold. All statistical analyses were conducted using IBM SPSS software (SPSS 25), and a *p*-value <0.05 was considered statistically significant.

## Results

### Study design and patient characteristics

This study was designed to investigate the clinical, biochemical, and diagnostic profiles of CKD patients with and without CAC. A total of 368 CKD patients were enrolled, comprising 216 with CAC and 152 without CAC (NCAC), as confirmed by CTA. Demographic, clinical, and biochemical parameters were systematically analyzed to identify key differences between the two groups (Table 1). The demographic and clinical characteristics of CKD patients with CAC (*n* = 216) and those without CAC (NCAC) (*n* = 152) revealed significant differences. Patients in the CAC group were significantly older than those in the NCAC group (61.81 ± 9.36 vs. 48.24 ± 12.73 years, *p* < 0.001). Additionally, the CAC group exhibited a higher prevalence of smoking (45.8 vs. 23.7%, *p* < 0.001), alcohol consumption (48.6 vs. 35.5%, *p* = 0.014), and diabetes mellitus (29.2 vs. 15.1%, *p* = 0.002). Advanced CKD stages (stage 4 and 5) were more prevalent in the CAC group (*p* < 0.001). Other clinical parameters, such as body mass index, sex distribution, and hypertension, did not show significant differences. Biochemical analysis indicated elevated serum phosphorus levels in the CAC group (1.25 ± 0.22 vs. 1.08 ± 0.18 mmol/L, *p* < 0.001), while serum calcium, high-density lipoprotein, low-density lipoprotein, total cholesterol, triglycerides, and alkaline phosphatase levels were comparable. Furthermore, primary pathogenesis didn’t exhibit any significant differences between the two groups. However, we observed significant changes in urine albumin-creatinine ratio, eGFR, serum creatinine, Ca × P, serum uric acid, PTH and vitamin K2 levels. These findings underscore the distinct clinical profile of CKD patients with CAC.

**Table 1.** Demographic and clinical factors for chronic kidney disease (CKD) patients with coronary artery calcification (CAC) or not (NCAC).

	NCAC (*n* = 152)	CAC (*n* = 216)	*p* value
Age (years)	48.24 ± 12.73	61.81 ± 9.36	<0.001
Body mass index (kg/m^2^)	23.69 ± 3.92	24.15 ± 4.17	0.383
Sex			
Male	92 (60.5%)	135 (62.5%)	0.744
Female	60 (39.5%)	81 (37.5%)	
Smoking			
Yes	36 (23.7%)	99 (45.8%)	<0.001
No	116 (76.3%)	117 (54.2%)	
Drinking			
Yes	54 (35.5%)	105 (48.6%)	0.014
No	98 (64.5%)	111 (51.4%)	
Diabetes mellitus			
Yes	23 (15.1%)	63 (29.2%)	0.002
No	129 (84.9%)	153 (70.8%)	
Hypertension			
Yes	107 (70.4%)	164 (75.9%)	0.279
No	45 (29.6%)	52 (24.1%)	
Stage of CKD			
1	56 (36.8%)	27 (12.5%)	<0.001
2	39 (25.7%)	42 (19.4%)	
3	28 (18.4%)	50 (23.1%)	
4	18 (11.8%)	58 (26.9%)	
5	11 (7.2%)	39 (18.1%)	
Primary pathogenesis			
Diabetic nephropathy	21 (13.8%)	51 (23.6%)	0.138
Primary glomerulonephritis	65 (42.8%)	80 (37.0%)	
Hypertensive nephropathy	44 (29.0%)	58 (26.9%)	
Polycystic kidneys	12 (7.9%)	19 (8.8%)	
Others	10 (6.6%)	8 (3.7%)	
UACR			
Normal	61 (40.1%)	52 (24.1%)	0.001
Abnormal	91 (59.9%)	164 (75.9%)	
HDL-C (mmol/L)	1.32 ± 0.61	1.28 ± 0.64	0.511
LDL-C (mmol/L)	3.67 ± 0.98	3.72 ± 1.05	0.647
TC (mmol/L)	4.33 ± 1.14	4.51 ± 1.25	0.283
TG (mmol/L)	1.52 ± 0.69	1.61 ± 0.72	0.317
Serum calcium (mmol/L)	2.24 ± 0.26	2.26 ± 0.29	0.405
Serum phosphorus (mmol/L)	1.08 ± 0.18	1.25 ± 0.22	<0.001
Serum ALP (U/L)	106.62 ± 39.81	113.29 ± 41.58	0.162
eGFR (mL/min・1.73 m^2^)	71.18 ± 19.09	56.47 ± 18.25	<0.001
Scr (μmol/L)	95.73 ± 29.17	119.36 ± 37.54	<0.001
Serum uric acid (μmol/L)	355.89 ± 90.12	394.27 ± 102.46	<0.001
Ca × P (mg^2^/dL^2^)	29.86 ± 5.24	35.14 ± 5.79	<0.001
Serum PTH (pg/mL)	115.38 ± 28.05	140.52 ± 35.48	<0.001
Serum vitamin K2 (ng/mL)	1.13 ± 0.39	0.73 ± 0.34	<0.001

The data were presented as mean ± SD or n (percentage). The comparisons of data between the NCAC and CAC groups were done by Mann Whitney test or Unpaired Student’s t-test with Welch’s correction or Fisher’s exact test or Chi-square test. ALP: alkaline phosphatase, eGFR: estimated glomerular filtration rate, Scr: serum creatinine, UACR: urine albumin-creatinine ratio.

### Increased Ca × P and PTH levels, and decreased vitamin K2, are associated with CAC in CKD

**Figure 1.**
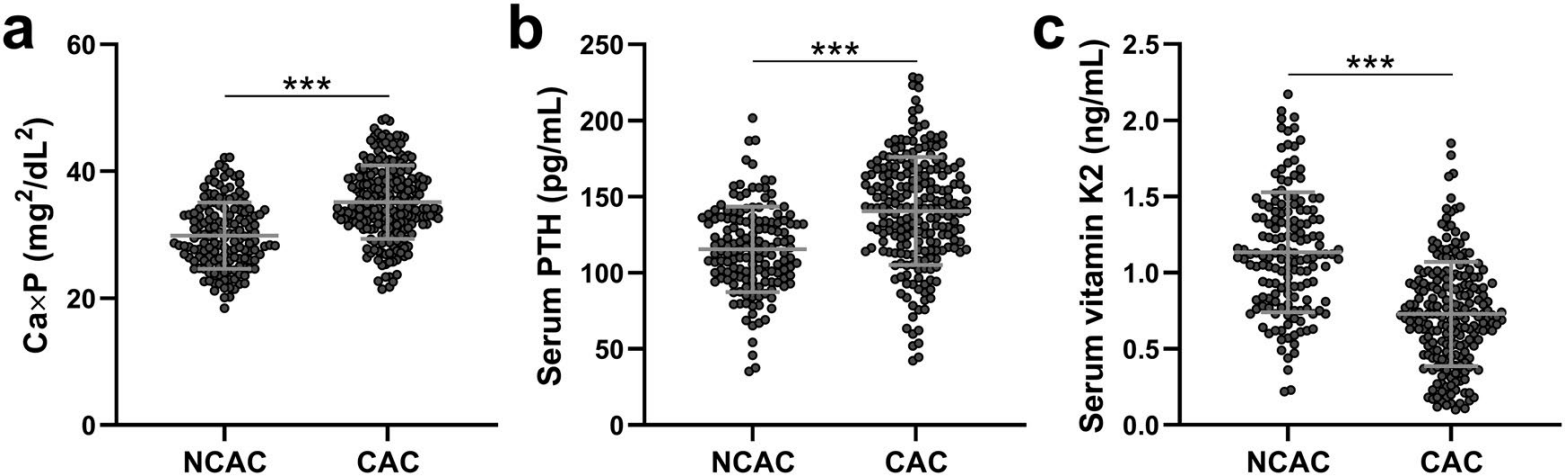
Comparisons of serum calcium-phosphorus product (Ca × P, a), PTH (b), vitamin K2 (c) between chronic kidney disease (CKD) patients with coronary artery calcification (CAC, *n* = 216) or not (NCAC, *n* = 152). The data were presented as mean ± SD. **p < 0.001 by Unpaired Student’s t-test with Welch’s correction.

Building on these clinical findings, we further investigated biochemical differences between CAC and NCAC groups (Figure 1). Patients with CAC had significantly elevated serum Ca × P (Figure 1a) and PTH levels (Figure 1b) compared to the NCAC group. Conversely, vitamin K2 levels (Figure 1c) were markedly lower in CAC patients. These results suggest that imbalances in Ca × P, PTH, and vitamin K2 may play critical roles in the pathogenesis of CAC in CKD patients.

### Ca × P, PTH, and vitamin K2 are significantly correlated in CAC patients

**Figure 2.**
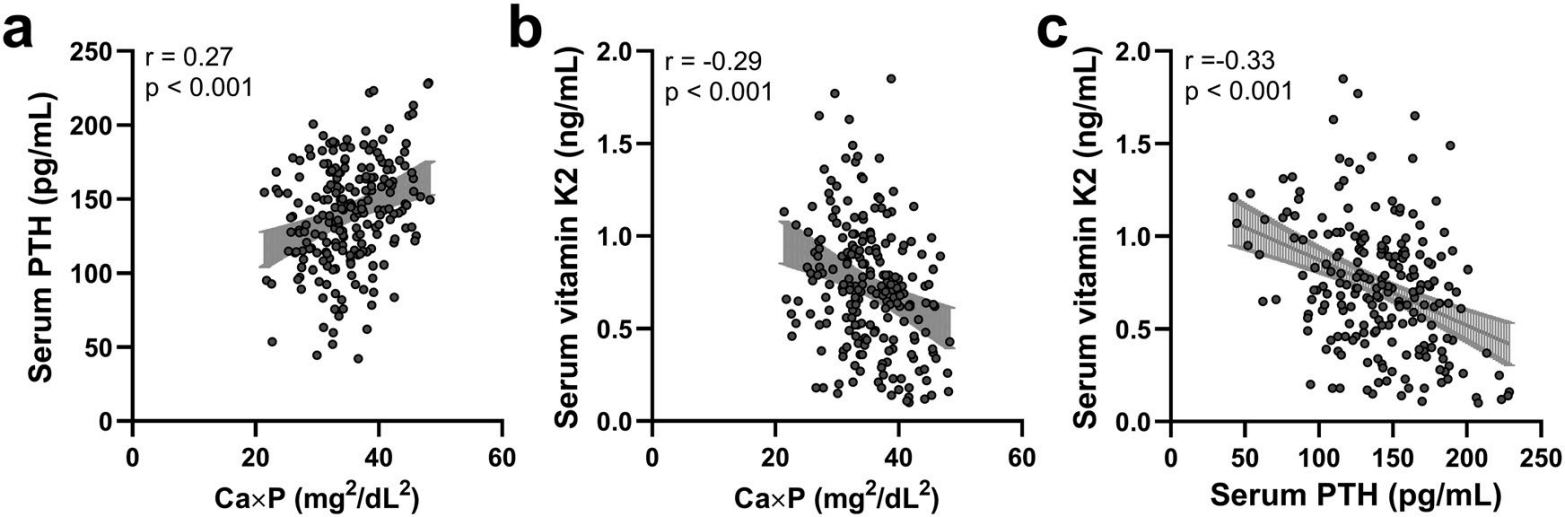
Pearson correlation analysis of serum Ca × P with PTH (a, *r* = 0.27 (0.14 to 0.39), *p* < 0.001), Ca × P with vitamin K2 (b, *r* = −0.29 (−0.42 to −0.16), *p* < 0.001) and PTH with vitamin K2 (c, r = −0.33 (−0.45 to −0.21), *p* < 0.001) in CKD patients with coronary artery calcification (CAC, *n* = 216).

To explore the relationships between these biomarkers, Pearson correlation analyses were conducted in the CAC group (Figure 2). A significant positive correlation was identified between Ca × P and PTH (Figure 2a, *r* = 0.27 (0.14 to 0.39, *p* < 0.001), indicating that higher Ca × P levels are associated with increased PTH levels. In contrast, Ca × P and vitamin K2 were negatively correlated (Figure 2b, *r* = −0.29 (−0.42 to −0.16), *p* < 0.001), suggesting that elevated Ca × P levels are associated with lower vitamin K2 concentrations. Similarly, PTH was inversely correlated with vitamin K2 (Figure 2c, *r* = −0.33 (−0.45 to −0.21), *p* < 0.001). These findings highlight the complex interplay among these markers, which may contribute to the progression of CAC in CKD patients.

### CAC severity positively correlates with Ca × P and PTH, and negatively with vitamin K2

**Figure 3.**
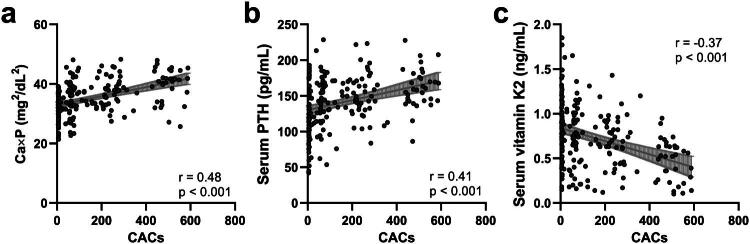
Spearman correlation analysis of coronary artery calcification score (CACs) with serum Ca × P (a, *r* = 0.48 (0.37 to 0.58), *p* < 0.001), PTH (b, *r* = 0.41 (0.29 to 0.52), *p* < 0.001), vitamin K2 (c, r = −0.37 (−0.49 to −0.25), *p* < 0.001) in CKD patients with coronary artery calcification (CAC, *n* = 216).

Having established significant correlations among the biochemical markers, we next examined their association with the severity of CAC, as measured by the CAC score (Figure 3). Both Ca × P (Figure 3a, *r* = 0.48 (0.37 to 0.58), *p* < 0.001) and PTH (Figure 3b, *r* = 0.41 (0.29–0.52), *p* < 0.001) were positively correlated with CAC severity, suggesting that higher levels of these markers are linked to more extensive calcification. In contrast, vitamin K2 levels negatively correlated with CAC severity (Figure 3c, *r* = −0.37 (−0.49 to −0.25), *p* < 0.001), indicating a potential protective role of vitamin K2 in mitigating calcification progression. These findings underscore the clinical utility of these biomarkers in assessing calcification severity.

### Combined biomarker analysis enhances CAC detection accuracy in CKD patients

**Figure 4.**
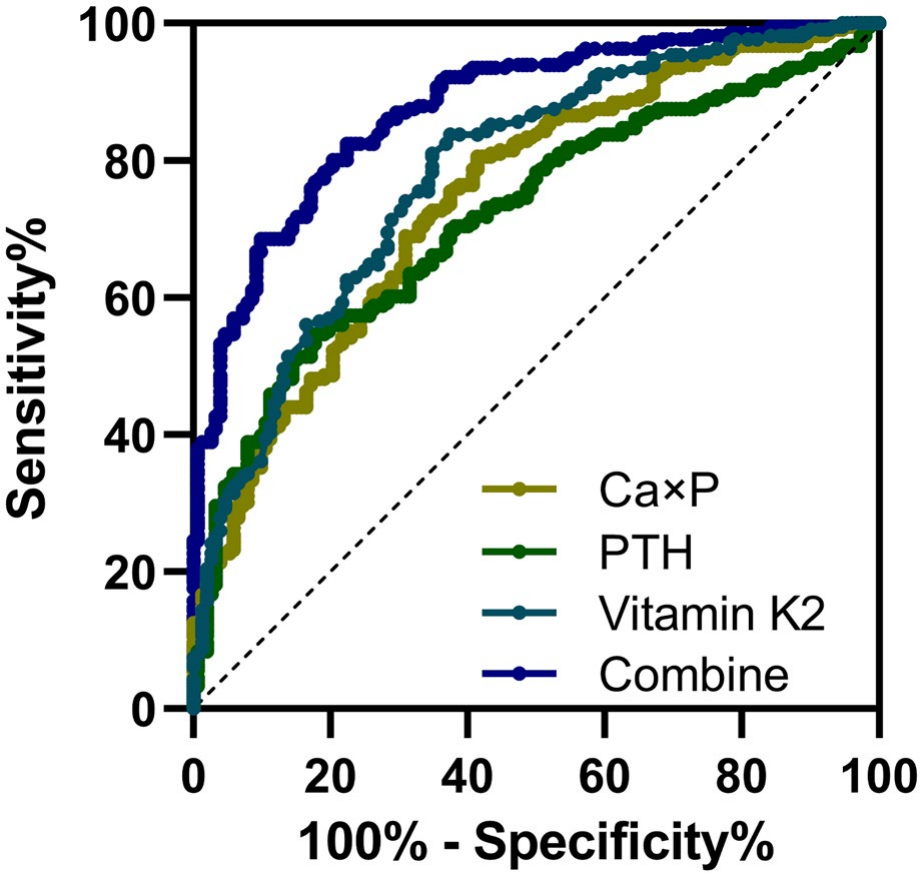
ROC analysis for diagnostic values of serum calcium-phosphorus product (Ca × P), PTH, vitamin K2 and their combine test for coronary artery calcification (CAC) in chronic kidney disease (CKD) patients.

Finally, we assessed the diagnostic performance of Ca × P, PTH, vitamin K2, and their combined use in detecting CAC in CKD patients using receiver operating characteristic (ROC) analysis (Table 2, Figure 4). Each biomarker exhibited significant diagnostic value individually, with Ca × P achieving an area under the curve (AUC) of 0.75 (95% CI: 0.70–0.80) and PTH achieving an AUC of 0.72 (95% CI: 0.67–0.77) (Figure 4). Vitamin K2 demonstrated the highest diagnostic performance among individual markers, with an AUC of 0.78 (95% CI: 0.74–0.83), sensitivity of 80.56%, and specificity of 65.13% at a cutoff value of 0.99 ng/mL. The combined model, which included Ca × P, PTH, and vitamin K2, significantly enhanced diagnostic accuracy, achieving an AUC of 0.88 (95% CI: 0.84–0.91), sensitivity of 82.41%, and specificity of 77.63%. These results demonstrate that combining multiple biomarkers provides a more robust strategy for diagnosing CAC in CKD patients.

**Table 2.** Diagnostic values of serum calcium-phosphorus product (Ca × P), PTH, vitamin K2 and their combine test for coronary artery calcification (CAC) in chronic kidney disease (CKD) patients.

	Cut off	AUC (**95% CI**)	p	Sensitivity (%)	Specificity (%)	Youden index
Ca × P	30.72 mg^2^/dL^2^	0.75 (0.70 to 0.80)	<0.001	80.56	58.55	0.39
PTH	138.60 pg/mL	0.72 (0.67 to 0.77)	<0.001	54.63	82.24	0.37
Vitamin K2	0.99 ng/mL	0.78 (0.74 to 0.83)	<0.001	80.56	65.13	0.46
Combine*	–	0.88 (0.84 to 0.91)	<0.001	82.41	77.63	0.60

CI: confidence interval.

* COMPUTE Combine = 0.167 × Ca × *p* + 0.024 × PTH–2.772 × Vitamin K2.

To verify the independent associations of serum Ca × P, PTH, and vitamin K2 with CAC occurrence while controlling for potential confounding factors, we performed multivariate logistic regression analyses, with detailed results summarized in Table 3. Two models were constructed: an unadjusted model including only the core biomarkers, and an adjusted model further incorporating confounders with *p* < 0.05 in Table 1. In the unadjusted model, all three core biomarkers showed significant associations with CAC. Serum Ca × P (OR = 1.41, 95%CI: 1.23–1.61, *p* < 0.001) and PTH (OR = 1.33, 95%CI: 1.15–1.53, *p* < 0.001) were positively associated with CAC risk, while serum vitamin K2 was negatively associated (OR = 0.58, 95%CI: 0.48–0.70, *p* < 0.001), consistent with our initial findings. After adjusting for the aforementioned confounders, the association strength of the core biomarkers showed slight but non-substantial reductions, confirming their stable independent relationships with CAC. Serum Ca × P remained a robust independent risk factor (OR = 1.35, 95%CI: 1.18–1.54, *p* < 0.001), serum PTH still correlated significantly with increased CAC risk (OR = 1.27, 95%CI: 1.10–1.47, *p* = 0.001), and serum vitamin K2 retained its independent protective effect (OR = 0.63, 95%CI: 0.52–0.76, *p* < 0.001). Among the adjusted variables, age, smoking history, diabetes mellitus, advanced CKD stages (stage 4 and stage 5), serum creatinine, serum uric acid, and abnormal UACR were independently associated with CAC occurrence. Collectively, these results validate that the three core biomarkers are robust independent predictors of CAC in CKD patients, supporting their rational inclusion in the diagnostic model.

**Table 3.** Results of multivariate logistic regression analysis.

Variable	Unadjusted model		Adjusted model	
	OR (95%CI)	*p* value	OR (95%CI)	*p* value
Core independent variables				
Serum Ca × P (mg²/dL²)	1.41 (1.23–1.61)	<0.001	1.35 (1.18–1.54)	<0.001
Serum PTH (pg/mL)	1.33 (1.15–1.53)	<0.001	1.27 (1.10–1.47)	0.001
Serum vitamin K2 (ng/mL)	0.58 (0.48–0.70)	<0.001	0.63 (0.52–0.76)	<0.001
Adjusted variables				
Age (years)	–	–	1.08 (1.03–1.14)	0.007
Smoking history (Yes = 1)	–	–	1.59 (1.16–2.95)	0.005
Alcohol consumption (Yes = 1)	–	–	1.37 (1.04–2.29)	0.026
Diabetes mellitus (Yes = 1)	–	–	2.03 (1.28–3.22)	0.003
CKD Stage (Ref = Stage 1)	–	–		
Stage 2	–	–	1.18 (0.65–2.14)	0.587
Stage 3	–	–	1.56 (0.86–2.83)	0.143
Stage 4	–	–	2.89 (1.55–5.39)	0.001
Stage 5	–	–	3.72 (1.88–7.36)	<0.001
Serum P (mmol/L)	–	–	1.15 (0.96–1.38)	0.121
Serum creatinine (μmol/L)	–	–	1.01 (1.00–1.02)	0.028
Serum uric acid (μmol/L)	–	–	1.003 (1.001–1.005)	0.006
UACR (Abnormal = 1)	–	–	1.78 (1.16–2.73)	0.008
Model fit	*χ*² = 68.72, *p* < 0.001; AUC = 0.79		*χ*² = 95.36, *p* < 0.001; AUC = 0.85	

Confounders included in the adjusted model are all variables with *p* < 0.05 in Table 1; OR values for continuous variables represent the “risk ratio of CAC occurrence for each 1-unit increase in the variable”; model fit was evaluated using the likelihood ratio *χ*² test and AUC value.

## Discussion

This study provides comprehensive insights into the clinical, biochemical, and diagnostic differences between CKD patients with and without CAC. The findings demonstrate significant elevations in Ca × P and PTH, alongside reductions in vitamin K2 levels in patients with CAC compared to those without CAC. These results highlight the critical roles of disrupted calcium-phosphorus metabolism, hyperparathyroidism, and vitamin K2 deficiency in the pathogenesis of CAC in CKD patients.

The elevated Ca × P and PTH levels observed in CAC patients align with previous studies showing that mineral metabolism abnormalities are significant contributors to vascular calcification in CKD [16]. Elevated Ca × P promotes osteogenic differentiation of vascular smooth muscle cells, leading to calcium deposition in arterial walls, while secondary hyperparathyroidism exacerbates calcium-phosphorus imbalance and induces arterial calcification [17,18]. The inverse relationship between vitamin K2 and CAC severity is consistent with its known role in activating MGP, a calcification inhibitor [19]. Vitamin K2 deficiency results in insufficient MGP activation, leaving arteries susceptible to calcium accumulation, particularly in CKD patients. Furthermore, vitamin K2 may exert a protective effect against CAC through multiple biological pathways. Adequate dietary intake of K2—especially from fermented foods—supports activation of MGP. Conversely, medications such as warfarin can impair MGP activation, potentially accelerating CAC progression. Gut microbiota also contribute to endogenous K2 synthesis, and dysbiosis in CKD patients may reduce this capacity. Together, these factors suggest that both nutritional and pharmacological modulation of K_2_ status could influence vascular health.

Our correlation analyses provide further evidence of the interplay between these biomarkers. The positive correlation between Ca × P and PTH reflects the well-established relationship between hyperphosphatemia and secondary hyperparathyroidism in CKD. Furthermore, the negative correlations of Ca × P and PTH with vitamin K2 levels underscore the multifactorial mechanisms underlying vascular calcification. These findings are consistent with previous research emphasizing the importance of addressing multiple pathways to mitigate vascular calcification in CKD [3,8,9].

The severity of CAC, as measured by CAC scores, showed significant positive correlations with Ca × P and PTH and a negative correlation with vitamin K2. These relationships suggest that these biomarkers can serve as reliable indicators of calcification progression in CKD patients. Compared to earlier studies that primarily focused on single biomarkers, our combined model demonstrated superior diagnostic accuracy, achieving an AUC of 0.88. This result underscores the potential for integrating multiple biomarkers into routine clinical practice to enhance risk stratification and guide targeted therapeutic interventions.

Clinically, these findings have important implications for managing CAC in CKD patients. While calcium-phosphorus metabolism and PTH regulation are already therapeutic targets in CKD management, our results highlight the potential value of incorporating vitamin K2 supplementation as an adjunctive therapy. Previous studies have shown that vitamin K2 supplementation can reduce vascular calcification in CKD patients, suggesting a novel strategy for mitigating cardiovascular risks in this population [5]. Additionally, combining multiple biomarkers, as demonstrated in this study, offers a more robust approach for early detection of CAC and personalized treatment planning [20].

One important issue worthy of noting is the exclusion of 25-hydroxyvitamin D and calcitonin from this study’s analysis. Measurements of 25-hydroxyvitamin D and calcitonin are typically performed on a case-by-case basis—such as when vitamin D deficiency or atypical bone metabolism is suspected. As a result, among the 368 patients included, only a small subset had records for these two biomarkers, with data completeness below 30%, which does not meet the criteria for complete clinical documentation required for analysis. In theory, 25-hydroxyvitamin D may influence serum calcium levels and thus be associated with CAC, hence its potential modulatory effect on the “Ca × P–PTH–CAC” relationship cannot be entirely ruled out. However, this study has partially mitigated such confounding through statistical adjustment for variables such as CKD stage and serum phosphorus. On the other hand, calcitonin’s core function is to inhibit bone resorption and reduce the release of calcium from bone stores. In theory, it may influence vascular calcification by modulating calcium metabolism. Nonetheless, in CKD patients, its regulatory priority is substantially lower compared to PTH.

However, several limitations of this study must be acknowledged. First, the retrospective design may introduce selection bias and limit causal inferences. Second, the cross-sectional nature of the data prevents us from assessing the temporal relationships between biomarker levels and CAC progression. Longitudinal studies are needed to validate our findings and evaluate their predictive value over time. Third, this study was conducted in a single center, which may limit the generalizability of the results to broader CKD populations with diverse demographic and clinical characteristics. Fourth, subgroup analyses could have been incorporated to enhance research depth, and we plan to incorporate such analyses in future prospective studies with larger sample sizes. In addition, given the cross-sectional design of the study, conclusions were merely correlative rather than causal inference between the biomarkers and CAC. Finally, we were unable to perform restricted cubic spline (RCS) analysis to verify the dose-response relationships between core variables and CAC occurrence due to the current resource of analysis tools. The absence of RCS analysis limits the precise description of quantitative trends in how biomarker level changes modulate CAC risk. This may hinder the identification of optimal clinical intervention thresholds for these biomarkers.

## Conclusions

In conclusion, this study underscores the significant correlation of Ca × P, PTH, and vitamin K2 with the development and severity of CAC in CKD patients. The combined use of these biomarkers may potentially enhance diagnostic accuracy of CAC. These findings pave the way for future research on integrating biomarker-based strategies into clinical practice and exploring novel therapeutic interventions, such as vitamin K2 supplementation, to improve cardiovascular outcomes in CKD patients.

## Data Availability

The data that support the findings of this study are available from the corresponding author, [C.C], upon reasonable request.
